# Developing Best Practices for Inclusion in fNIRS Research: Equity for Participants With Afro‐Textured Hair

**DOI:** 10.1002/dev.70134

**Published:** 2026-02-05

**Authors:** Abria S. Simmons, Gavkhar Abdurokhmonova, Ellie K. Taylor‐Robinette, Rachel R. Romeo

**Affiliations:** ^1^ University of Maryland College Park Maryland USA

**Keywords:** Afro‐textured hair, best practices, fNIRS, inclusion

## Abstract

Functional near‐infrared spectroscopy (fNIRS) is a popular optical neuroimaging method; however, participants with Afro‐textured (i.e., dark, coarse, curly) hair are often excluded due to difficulty obtaining sensor–scalp contact. Grounded in lived experience and sociocultural literature, we aimed to develop and evaluate culturally responsive best practices for participant interaction and hair preparation to increase Black participant inclusion in fNIRS research. First, we developed an intake survey, guidelines for researcher staffing and training, and a suite of customizable hair preparation techniques that prioritize participant comfort and hair integrity. We then evaluated these techniques with 19 Black participants (11 adults, eight children) with varying hair types/styles; methods included braiding cornrows around the intended optode montage, using gels and clips to part hair, and various ways of increasing tension to promote sensor–scalp contact. On average, signal quality improved by 50%, with the greatest improvements in anterior regions. While signal quality was not perfect, it was generally improved to the point of acceptability and inclusion in a racially/ethnically diverse dataset (with hair type/color as covariates). We conclude with recommendations for increasing awareness of racial bias in neuroimaging, greater diversity in research teams, and a more inclusive approach for working with diverse populations.

## 1 Introduction

Functional near‐infrared spectroscopy (fNIRS) is an optical, mobile, and motion‐tolerant neuroimaging method well‐suited for brain analyses in naturalistic, real‐world settings, thus providing wide opportunities to collect ecologically valid neuroimaging data. However, the optical properties of near‐infrared light and the design of fNIRS hardware are often incompatible with hair of darker color and coilier texture (e.g., Afro‐textured hair). Accordingly, many fNIRS research findings have been based on samples that exclude Black participants with Afro‐textured hair (Kwasa et al. 2023). Thus, the present study aimed to develop a series of culturally responsive, participant‐centered best practices for fNIRS participants (adults and children) with Afro‐textured hair and to empirically evaluate whether these procedures improve signal quality and, ultimately, participant inclusion.

### 1.1 fNIRS as a Research Tool

fNIRS is a noninvasive, cap‐based, optical neuroimaging technique utilized to index brain activation via the hemodynamic response in cortical regions of the brain. fNIRS sources shine harmless near‐infrared light into the skull that penetrates the outer layers of the brain's cortex a few centimeters deep. This near‐infrared light then scatters and reflects, and strategically placed detectors (approximately 3 cm from the sources) measure the light that is reflected back out to the scalp through a “banana” shaped path. Because oxygenated and deoxygenated blood have different hemodynamic properties, and thus reflect different amounts of light, these features allow estimation of the intensity of brain activity taking place in specific measured regions of interest (Ferrari and Quaresima 2012; Quaresima and Ferrari 2019; Scholkmann et al. 2014).

Scholars have increasingly employed fNIRS as a tool over the past decade because of its many advantages, including lower cost, portability, and motion tolerance (relative to other techniques such as MRI and EEG). The portability of fNIRS invites the possibility of data collection in more naturalistic and ecologically valid non‐lab settings, which can increase the inclusion of participants facing barriers to participating in lab‐based research at a university or medical center (e.g., transportation issues, dislike of medical settings) (Arredondo 2021; Pinti et al. 2018). Additionally, the relative motion tolerance is well‐suited for individuals or populations who might struggle to sit perfectly still for long periods of time, such as young children and those with developmental or psychiatric disabilities (Blasi et al. 2019; Rahman et al. 2020; Wilcox and Bioindi 2015; Zhang and Roeyers 2019). However, research with fNIRS is not without technological limitations, as discussed below.

### 1.2 Features of Afro‐Textured Hair

**FIGURE 1:**
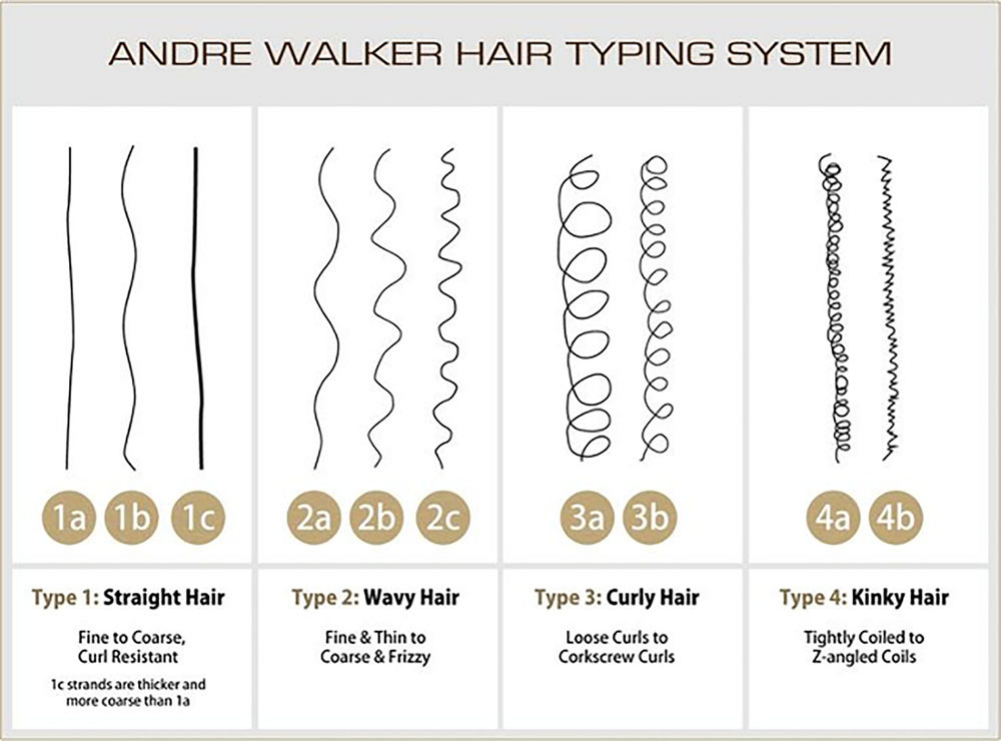
Andre Walker hair typing system (image from Women Health Info Blog [2017]).

“Afro‐textured” is a term used to describe features of the naturally curly hair common in people of African descent (henceforth referred to as Black). While people from all races can have curly hair, Afro‐textured hair is characterized by several features, including “type, texture, elasticity, porosity, density, and natural curl and shape” (Brown and Lemi 2021). Hair texture refers to the thickness of the hair strand; elasticity refers to the breakability of the hair; porosity refers to the hair's absorbency; density refers to number of strands per unit of scalp; and curl and shape refer to how bent or coiled each curl is, which can vary substantially across a single individual's scalp (Brown and Lemi 2021). While there are many classification systems for hair type, the most commonly used one in the Black hair community is the Andre Walker hair typing system (Figure 1), which groups hair types into one of four categories: Type 1, or straight hair; Type 2, or wavy hair; Type 3, or curly hair; and Type 4, or kinky hair (Divina Blk 2021; Trebilcock 2017). The majority of Black individuals have Type 3 or 4 hair, where Type 3 is described as having a “loopy” pattern in the shape of an “S,” where curls are defined and may have substantial volume, and Type 4 is described as visibly patterned with very tight curls (Ellis‐Hervey et al. 2016). While this hair‐typing system is not without criticism (e.g., Gaines et al. 2023), utilizing such a typology can be a helpful starting point for understanding features of Afro‐textured hair and the need for specialized approaches in neuroscience. Given the simplicity and familiarity of this system in the Black community, we use this system in communicating about hair type with participants before participating in neuroscience research.

### 1.3 Barriers to fNIRS Research for Participants With Afro‐Textured Hair

**FIGURE 2:**
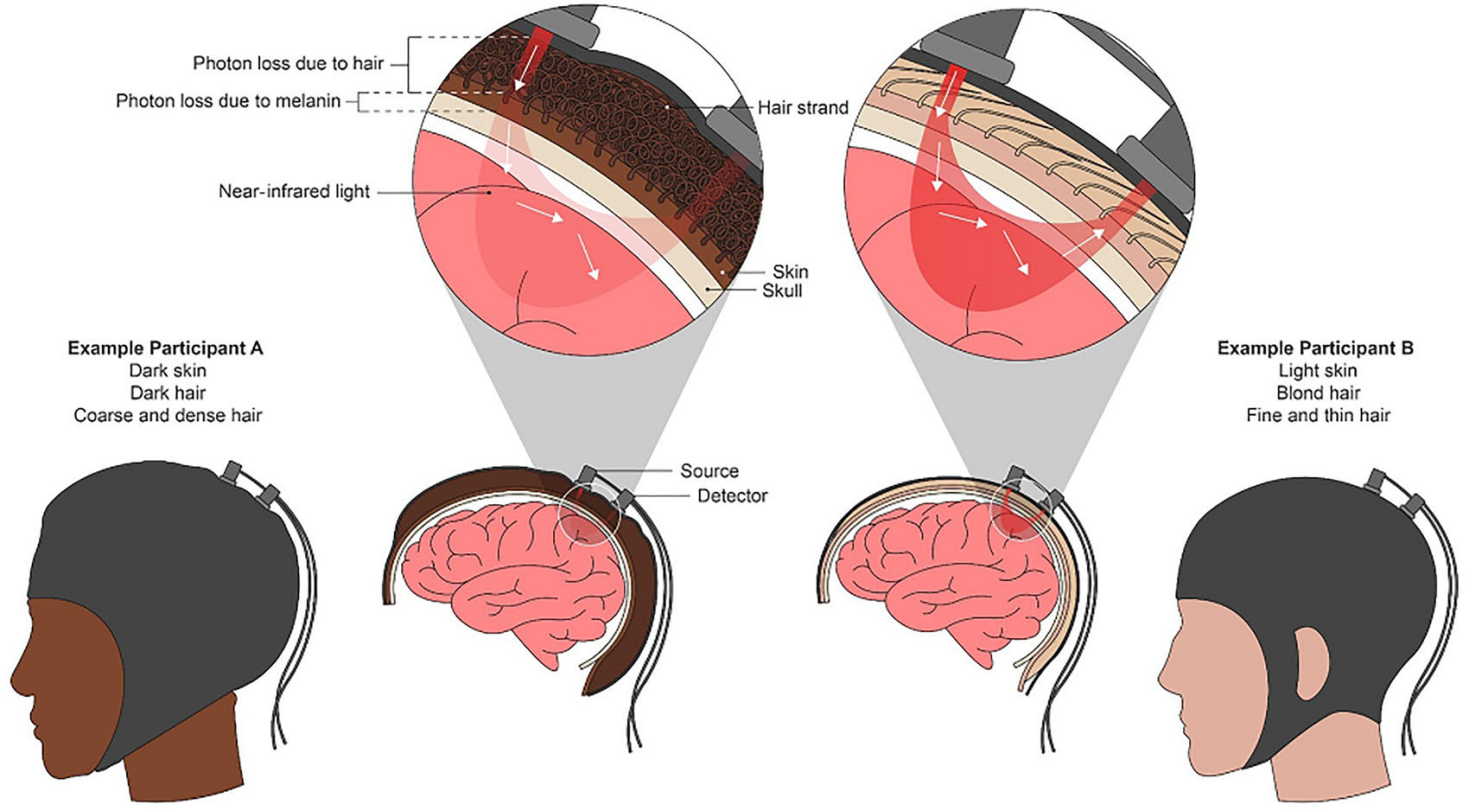
Illustration of fNIRS signal loss across different hair types, hair colors, and skin pigments. Reprinted with permission from Kwasa et al. (2023).

Because the fNIRS signal is light based, the optical properties of the hair, skin, and skull attenuate the signal quality (Figure 2). For individuals with fine, straight, light‐colored hair and lighter skin tones, it is relatively easy to achieve scalp–optode contact and, in most cases, usable signal quality. However, the data acquisition process is not as straightforward for participants of all hair and skin types.

One barrier to acquiring a strong fNIRS signal with Black participants is that the dense, coily, and thick nature of Afro‐textured hair can make it difficult for the optodes to penetrate through the hair and maintain strong, consistent contact with the scalp (optodes must stay securely in the same point on the scalp to collect data over time). Poor contact and optode instability may reduce the photon levels of the near‐infrared light and ultimately attenuate the signal present at the detectors. While many fNIRS systems automatically adjust the source brightness to accommodate for some amount of attenuation, it is essential to achieve as close and stable optode–scalp contact as possible. This typically requires some amount of hair preparation and manipulation, which most fNIRS hardware is not optimized for.

These physical realities and technological biases have led to systemic exclusion of participants with Afro‐textured hair in fNIRS and other neuroscientific research.^1^ However, the rapidly growing body of fNIRS research exhibits extremely limited reporting of demographic factors and other relevant contextual information about participants, as noted by two recent systematic reviews. Girolamo and colleagues reviewed 38 fNIRS studies and found that only 5% reported participant race and ethnicity, compared to 89% that reported gender and 100% that reported geographic location (Girolamo et al. 2022). Similarly, Kwasa and colleagues evaluated 87 fNIRS papers from 2017 to 2022 and found that a mere 2% reported race and ethnicity, 0% reported skin tone, and 3% reported hair type, compared to over 90% that reported participant gender (Kwasa et al. 2023). Given this systematic underreporting, it is difficult to fully quantify the level of exclusion based on race, ethnicity, skin tone, and hair type and texture in fNIRS research, though it is likely similar if not even greater than that seen with other neuroscientific modalities (Bradford et al. 2024; Choy et al. 2021; Etienne et al. 2020; Kwasa 2024; Louis et al. 2022; Parker and Ricard 2022).

Thus, there is a critical and immediate need for improving fNIRS research approaches to increase Black participant inclusion. While technological innovation is certainly needed (see Yücel et al. 2024), we instead focus on developing approaches that researchers can employ using *existing* fNIRS technology. However, it is essential to first consider several cultural realities in participant‐centered best practices.

### 1.4 Sociocultural Circumstances to Consider When Developing Best Practices for fNIRS Research With Afro‐Textured Hair

#### 1.4.1 Stigmatization of Afro‐Textured Hair and Protective Styles

Due to Eurocentric beauty standards and persistent anti‐Black racism, Afro‐textured hair faces significant stigmatization. “Texturism” is the hierarchical positioning of straight hair and looser curl patterns above tighter, kinkier ones (Dixon and Telles 2017; Rowe 2021; Shepherd 2018). “Good hair” is often characterized by straight or wavy textures that align with Eurocentric standards, while “bad hair” is labeled as tightly coiled and thick (Robinson 2011). Many Black individuals choose to chemically relax their hair to appear straight, despite the potential damage this may cause, because “natural” hair texture can be a barrier to social and professional acceptance (Ellis‐Hervey et al. 2016; Gray 2017; Khumalo et al. 2010; Yosso 2005). This stigmatization is so pervasive that in 23 US states, it remains legal to discriminate against Black individuals for employment and educational purposes based on their hair texture and style (CROWN Coalition and Dove 2025). Because of this reality, many Black individuals consider that wearing their hair naturally is a political statement that represents Black pride, despite ongoing media portrayals favoring Eurocentric hair standards.

Relatedly, many individuals with Afro‐textured hair wear their hair in protective hairstyles, such as cornrows, braids, twists, locs, Bantu knots, weaves, and wigs. These styles prevent breakage and promote hair health, and may include only the wearer's own hair or may also incorporate extensions or other additional hairpieces (Sandeen and Hancock 2024). These styles often require considerable time (a full day or more) and expense (up to several hundred dollars) to implement, leading many to wear them for weeks, months, or even years.

Thus, hair is deeply tied to identity and culture within the Black community, so researchers should respect participants’ styling choices and never ask individuals to relax their hair or remove protective styles solely for research participation (Thompson 2008). Such requests are likely to be seen as ignorant and offensive, and reduce chances of inclusion.

#### 1.4.2 The Role of Wash Day and Using Black Hair Care Products

For those with non‐Afro‐textured hair, washing one's hair is most often a straightforward part of their daily, every other day, or weekly routine. Even with long, thick hair, the hair washing process rarely takes more than half an hour to complete. Furthermore, convenience stores, drug stores, and grocery stores stock a wide variety of applicable hair care products that can be used.

In contrast, for those with Afro‐textured hair, “wash day” refers to a scheduled hair‐washing day, because this process can take several hours or even days to complete, depending on the amount and type of hair, whether a style is being removed, and the type of new style being implemented. The wash day process often involves detangling, washing, deep conditioning, and styling, which needs to be conducted with hair care products designed specifically for Afro‐textured hair to avoid damage to the hair follicle. To learn more about wash day, we recommend the monograph *Wash Day: Passing on the Legacy, Rituals, and Love of Natural Hair* by Tomesha Faxio (Faxio 2023, 2024; White‐Grier 2024).

Preparation for fNIRS studies almost always requires manipulation and “messing up” of a participant's hair. While this may be a minor inconvenience for many participants, for those with Afro‐textured hair, these manipulations need to be planned in advance, so that participants know what to expect and can plan enough time to return their hair to the way they want it. Ideally, researchers can schedule fNIRS sessions either immediately before or after participants’ intended wash day, when they were already planning on taking out the existing hairstyles. Additionally, researchers should prepare Afro‐textured hair with products specifically designed for Afro‐textured hair that will not cause long‐term damage to the hair (see Supporting Information S1 for a suggested shopping list).

#### 1.4.3 Cultural Significance of the Hair Salon and Relationships With Barbers/Beauticians

A final consideration is the role of the hair salon or barbershop as something of a sacred place. These establishments function as places where community members can discuss challenging topics while celebrating relationships, resilience, and joy (Palmer et al. 2022; Solomon et al. 2004). Importantly, the loyalty between clients and hair care professionals runs extraordinarily deep, with relationships often lasting decades and extending beyond professional services. Stylists and barbers frequently become integrated into clients’ families, attending important events and sometimes even styling deceased clients’ hair for funerals. Given the deep trust and loyalty inherent in hair care relationships, participants with Afro‐textured hair may be hesitant to allow unknown researchers to manipulate their hair (Palmer et al. 2022). Combined with the history of stigmatization, participants may be understandably nervous, distrustful, and even alienated by well‐meaning but poorly informed researchers. This underscores the importance of developing culturally responsive approaches for fNIRS research and ideally, as described below, the inclusion of research team members who share similar values and positionality.

### 1.5 Prior Efforts Toward More Inclusive fNIRS Research

Promisingly, many scholars have recently published calls to action and specific recommendations to increase diversity, equity, and inclusion in human neuroscience, especially in EEG, which uses a cap and scalp‐based sensors similar to fNIRS (Adams et al. 2024; Etienne et al. 2020; Garcini et al. 2022; La Scala et al. 2023; Margolis et al. 2025; Mlandu et al. 2024; Murray et al. 2021; Richardson 2021; Webb et al. 2022; Wu et al. 2024). For example, Lietsel Richardson's “A Guide to Hair Preparation for EEG Studies” includes specific subject preparation recommendations, including style suggestions (braided crown, tight low bun, pigtail braids), and scheduling considerations, such as scheduling data collection to coincide with washday for removable styles or right before a retwist session for nonremovable styles (Richardson 2021). This may be especially relevant in pediatric populations, when investigators commonly reach out to families close to a child's birthday when they age into a new condition or project (Adams et al. 2024). However, birthdays and other major life events often coincide with special, and sometimes expensive, hairstyles being put in place (“birthday hair”) (Adams et al. 2024). These considerations underscore the importance of communication between the research team and research participants to maximize comfort and uptake.

Inclusive practices specifically for fNIRS research—and especially pediatric fNIRS—are much more limited. Although some researchers have recently discussed these and other equity‐related concerns with fNIRS (e.g., Cheaye and Rosen n.d.; Doherty et al. 2023; Eng 2024; Khan et al. 2012; Yücel et al. 2024), solutions have largely focused on hardware re‐design rather than specific best practices in participant interaction and hair preparation. Thus, we set out to develop participant‐centered best practices to improve inclusion of participants with Afro‐textured hair in fNIRS research. Rather than providing a simple, universally applicable solution, our work over the past several years has highlighted the importance of personalization and respect for the individual.

### 1.6 Objectives of the Present Study

Based on the literature reviewed above and grounded in the first author's lived experience, we aimed to develop best practices to improve fNIRS signal quality for participants with Afro‐textured hair, while prioritizing participant comfort, hair integrity, and cultural de‐stigmatization. Below, we describe the various methods we employed before, during, and after the visit. We then report quantitative improvements in signal quality, and finally, we conclude by providing recommended “best practices” for other researchers in this field.

## 2 Methods

### 2.1 Participants

Nineteen individuals with Afro‐textured hair (11 adults, eight children; eight male, 11 female) participated in the present study. Child participants (ages 3–5 years) were participating in a longitudinal study of cognitive and brain development across preschool ages, and thus, the present investigation took place as a part of their planned fNIRS session in which they completed touchscreen‐based tasks of language, theory of mind, and executive functioning skills. Adult participants were friends, family, and colleagues of author A.S. who were specifically invited to participate in this investigation. All participants identified as Black/African American with Afro‐textured hair of different styles. Table 1 details participants’ sex, hair type/texture, porosity, wash frequency, and day‐of‐visit hairstyle. Unfortunately, signal quality data for Participant C1 were lost and thus not included in analyses, but we leave their information in the table for completeness. All participants (or their parent/guardian) provided informed consent and were compensated $20/h.

**TABLE 1:** Information on participants’ hair texture, general washing routine, style on the day of testing, final intervention, and percent improvement in usable channels (i.e., green/excellent and yellow/acceptable combined, out of the total number of data channels).

ID	Sex (M/F)	Hair type/texture and porosity level	Frequency of hair washing	Day of visit hairstyle	Intervention location	Hair intervention	% improvement in usable channels
A1	F	4b medium	Every other week	Box Braids	Right side of the head only	Braided into larger cornrows (three box braids at a time)	Pre/post: NA Left/right: 5%
A2	F	3a low	Weekly	Box Braids	Right side of the head only	Strategic pinning of braids	Pre/post: 0% Left/right: −5%
A3	F	4b medium	Weekly, taking 2–3 h	Afro	Right side of the head only	Cornrows	Pre/post: 64% Left/right: 23%
A4	F	3b medium	Monthly, taking 5–8 h	Box Braids	Right side of the head only	Strategic pinning of braids and then cornrowing of braids	Pre/post: NA Left/right: 0%
A5	F	4c medium	Every 2–3 weeks	Afro	Right side of the head only	Cornrows	Pre/post: 111% Left/right: 36%
A6	F	4c high	Every 2–3 weeks	Mini two‐strand twists	Right side of the head only	Mini twists separated and braided into larger sections	Pre/post: 43% Left/right: 50%
A7	M	4c medium	Daily	Afro	Right side of the head only	Cornrows	Pre/post: 113% Left/right: 5%
A8	M	4b low	Twice a week	Locs	Right side of the head only	Locs strategically pinned away from optode locations	Pre/post: NA Left/right: 5%
A9	M	3b and 4c combo; low	Every 2 weeks	Afro	Right side of the head only	Cornrows	Pre/post: 150% Left/right: 55%
A10	M	4b low	Unknown	Afro	Right side of the head only	Cornrows	Pre/post: 19% Left/right: 59%
A11	F	4b medium	Every other week	Afro	Entire head	Cornrows done on the whole head	Pre/post: NA Left/right: 27%
C1^a^	F	3c low	Every 6–12 days	Study‐specific cornrows done at home by a parent	Entire head	Some cornrows left as is, some rebraided to fit measurements better	Pre/post: NA Left/right: N/A
C2	M	3b high	Unknown	Afro	Entire head	Short hair gelled down	Pre/post: NA Left/right: N/A
C3	M	3b medium	Weekly, on Wednesdays	Afro	Entire head	Partial cornrows to the front of the head, and crochet hooks for the rest	Pre/post: 20% Left/right: N/A
C4	M	4c low	Once a week	Afro	Entire head	Hair piled in midline and then moved with crochet hooks	Pre/post: NA Left/right: N/A
C5	M	4c low	Three to four times per week	Afro	Entire head	Whole head gelled and cornrowed	Pre/post: 17% Left/right: N/A
C6	F	4c low	Unknown	Afro	Entire head	Cornrows done on the whole head	Pre/post: 33% Left/right: N/A
C7	F	4c low	Unknown	Afro	Entire head	Cornrows done on the whole head	Pre/post: 33% Left/right: N/A
C8	F	4a	Every couple of weeks	Two‐strand twist pigtails	Entire head	Cornrows done on the whole head	Pre/post: 3% Left/right: N/A

*Note:* A = adult; C = child.

^a^ Excluded from analysis.

### 2.2 fNIRS System and Montage Specifications

**FIGURE 3:**
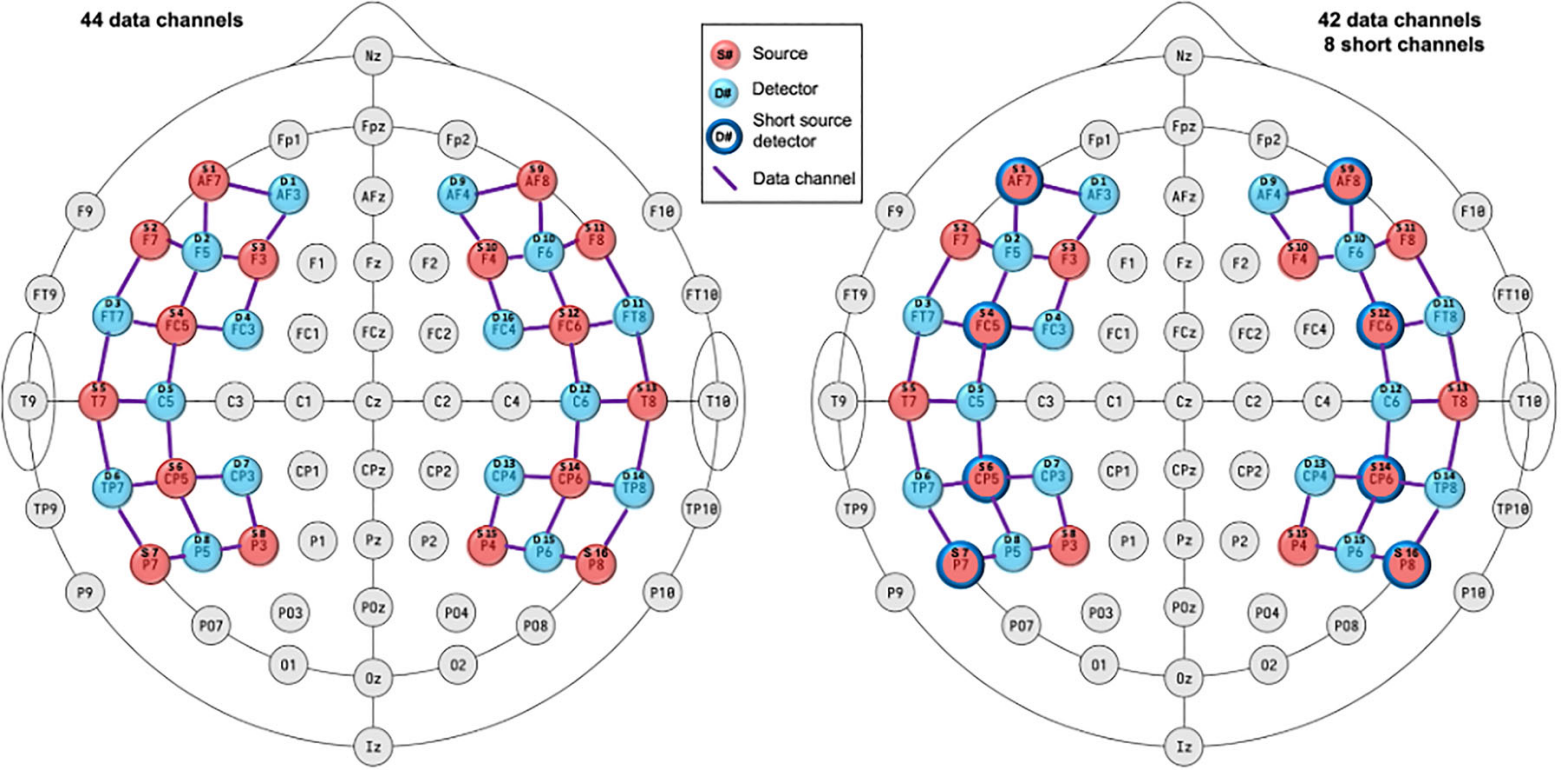
fNIRS montage. Sources are in red and detectors in blue, with the source or detector number at the top and the EEG location name at the bottom. Purple lines represent regular data channels, and dark blue circle outlines (right side) represent short‐distance detectors that each create an additional “short” data channel. The left image depicts the regular 44‐channel montage, while the right depicts a 42‐channel montage with eight short‐distance channels replacing detector 16 (FC4).

Data were acquired using a NIRx NIRSport2 mobile fNIRS system with 32 dual‐tip LED optodes (16 sources, 16 detectors). The montage was designed to cover brain regions associated with language, executive functioning, and social cognition, which includes bilateral prefrontal, superior/middle temporal, and temporoparietal regions (Figure 3, left). Ultimately, this created 44 topographic data channels of interest between adjacent sources and detectors (22 per hemisphere). Optodes were populated into the appropriately sized EasyCap, ranging in size from 52 cm (child participants) to 60 cm (adult male). The initial optode population used spring‐loaded tension level 1 on the forehead (anterior four optodes on each side), tension level 2 on the front/middle of the head (the next six optodes), and tension level 3 on the back of the head (posterior six optodes); this configuration was often altered (see below).

For 15 participants (including all seven children and eight adults), one detector (F4, in right superior frontal) was sacrificed and replaced with eight short‐distance detectors that were distributed evenly throughout the montage, whose purpose is to collect nonneural physiological signals for regressing out of the data from the primary channels. This left 42 long‐distance data channels, plus eight short‐distance data channels (Figure 3, right). Because there was less variation in the short‐distance channels, we primarily examined the improvement of the long‐distance data channels (see Supporting Information S2 and Figure S1 for short‐distance channels).

### 2.3 Signal Optimization

**FIGURE 4:**
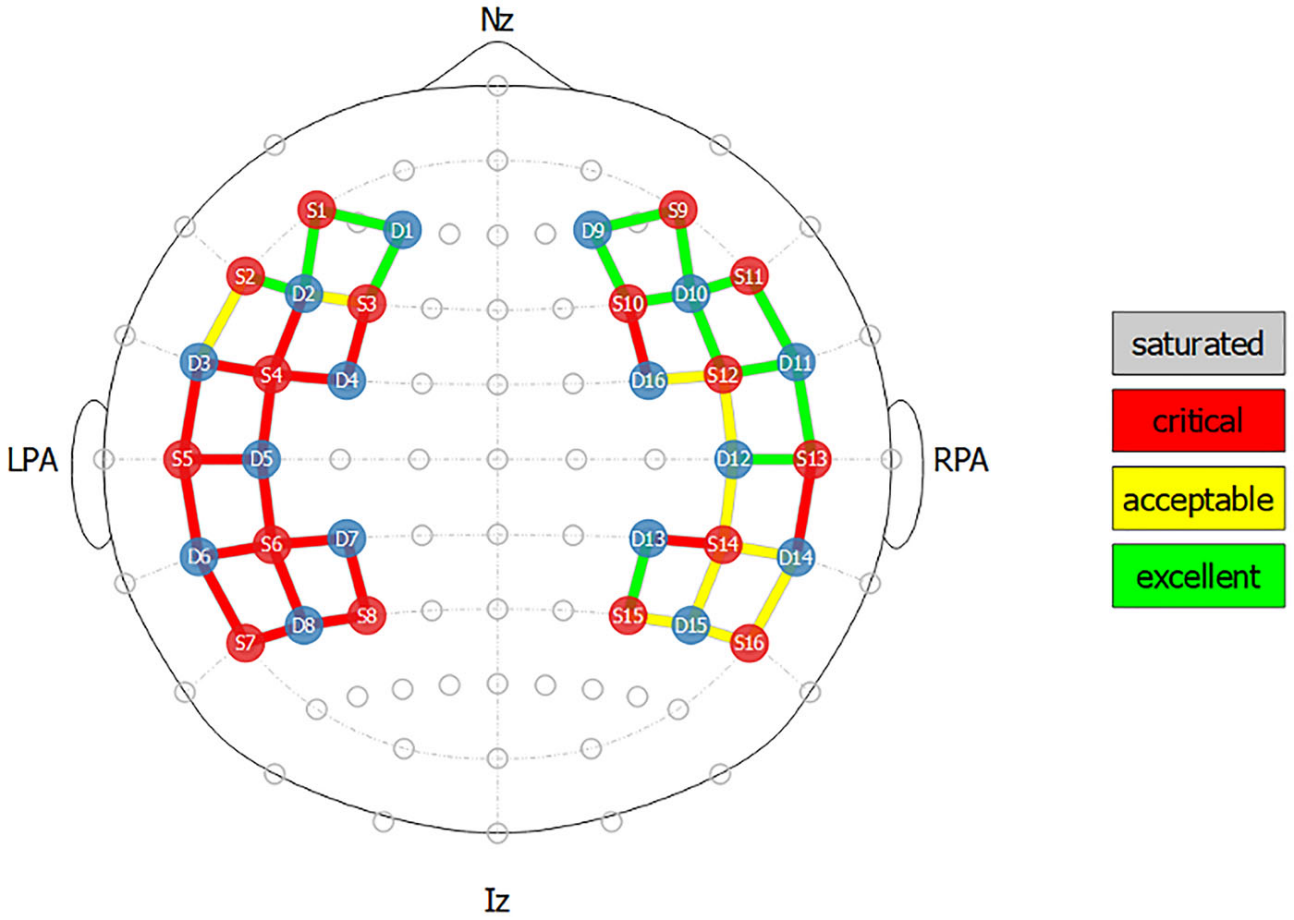
Sample fNIRS postintervention signal optimization on a Black adult participant showing the difference in signal quality between the left side (with no intervention/existing hairstyle) and the right side (undergone braiding/cornrowing intervention) of the head.

During the calibration process, the NIRSport2 device increases the brightness of each source in a stepwise manner (from 0% to 100%) until the optimal signal amplitude for each channel is obtained. The Aurora software then provides a stop‐light–colored rating system for each data channel to signal researchers which channels to improve before starting the experiment (Figure 4). Red indicates critical or poor signal quality; yellow indicates acceptable (but potentially improvable) signal quality; and green indicates excellent signal quality that needs no further improvement. The colored values are based on the raw voltage measured at each detector (red <0.5 mV; yellow = between 0.5 and 3 mV; green >3 mV). While these quantitative values are provided in real time during signal optimization, they change rapidly based on the physiological signal, while the coarser color categories are more stable and are typically the basis of researchers’ decisions to proceed. As stated in the Aurora user guide, “it is OK to proceed to recording with some yellow channels, but not advisable to proceed with red channels, as these will likely need to be excluded in later analysis for having a high coefficient of variation (C.V.), or noise” (NIRx Medical Technologies 2023, 25). Thus, we use the color categories as the primary outcome of interest.

### 2.4 Procedures

**FIGURE 5:**
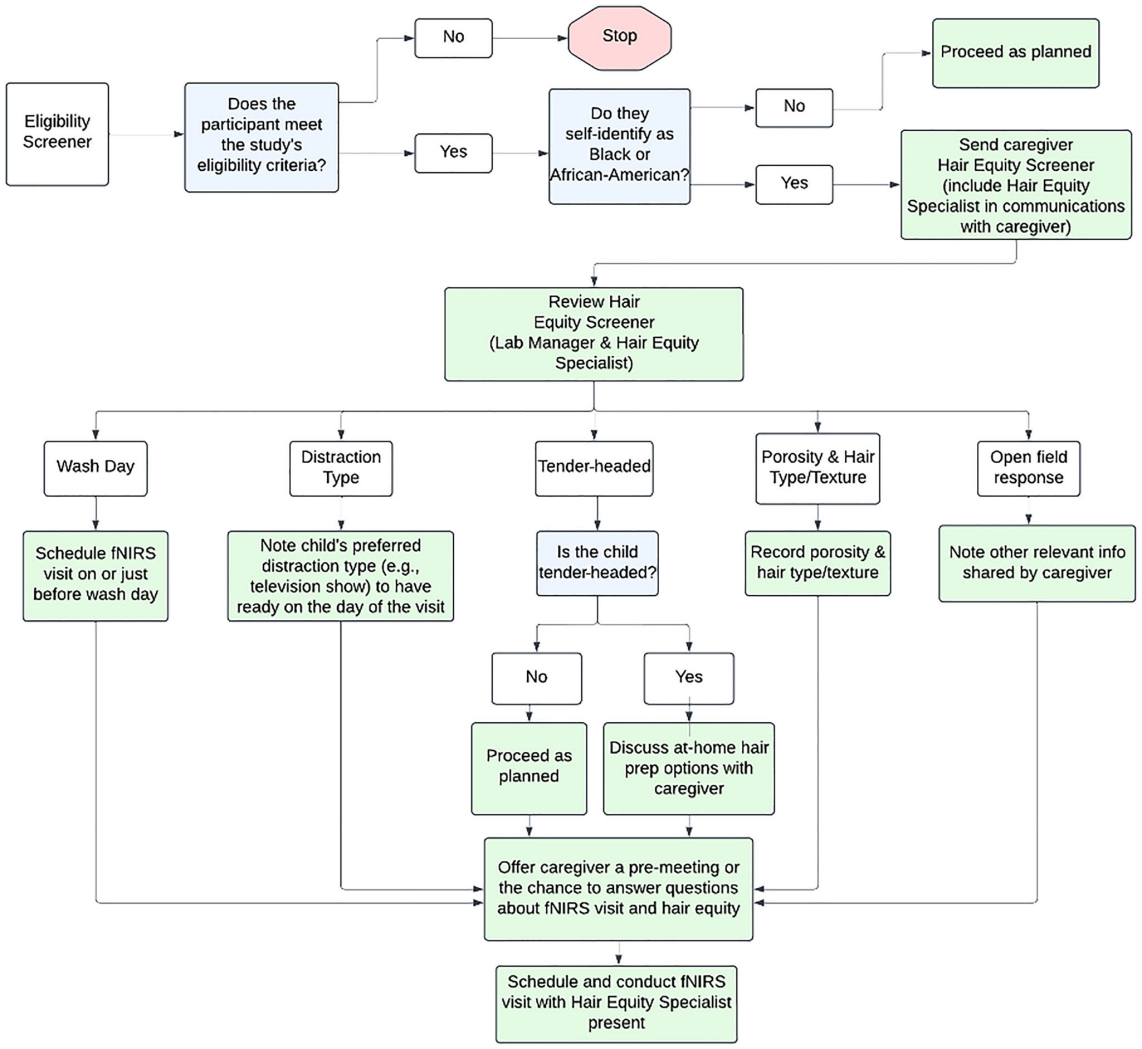
Flowchart of recruitment, eligibility screening, hair equity questionnaire, and scheduling.

**FIGURE 6:**
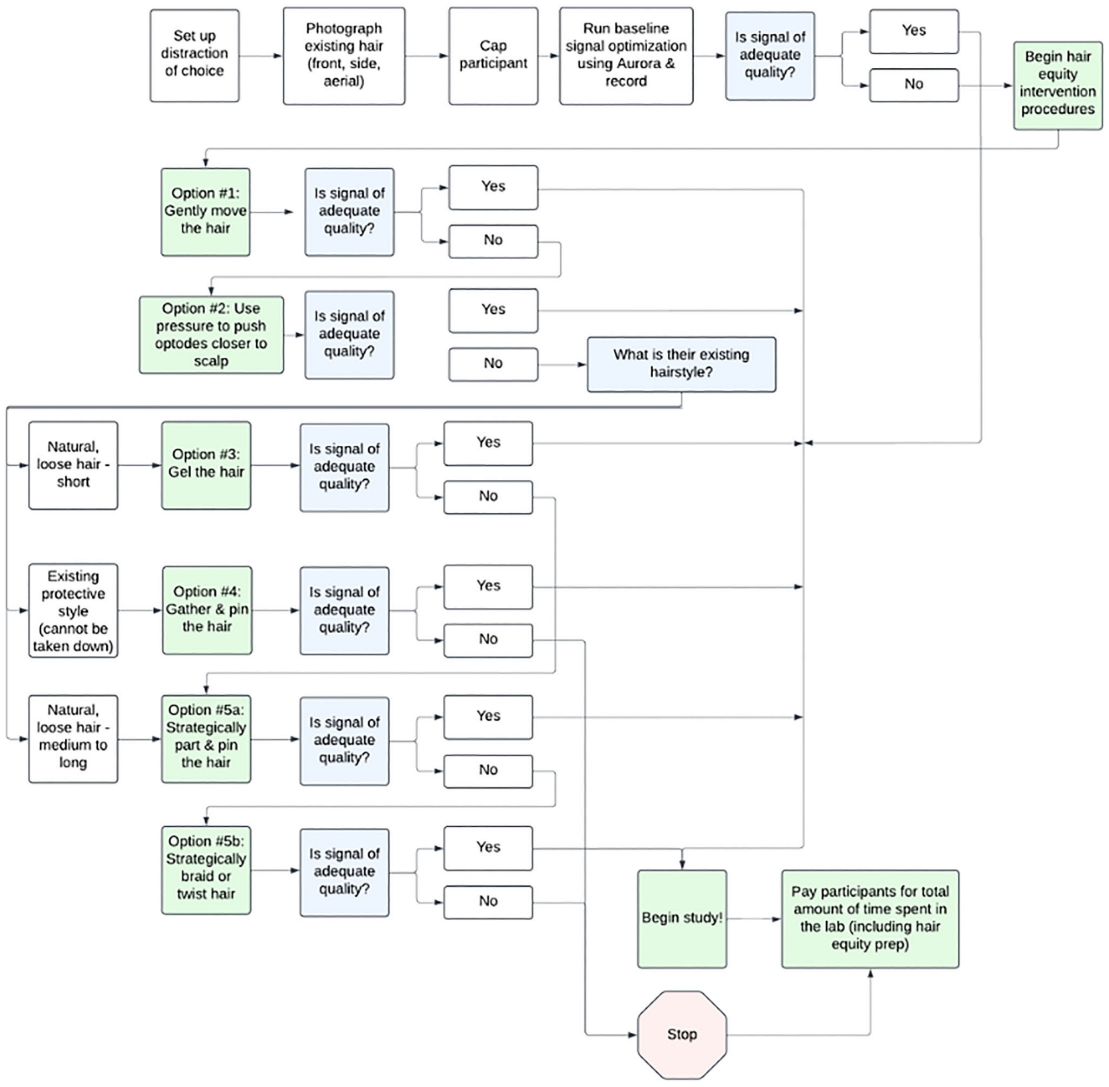
Flowchart of hair equity intervention options and procedures.

Below, we describe our procedures in three sections: activities before the visit, during the visit, and after the visit. We also provide two flowcharts to aid in understanding these procedures: Figure 5 describes the recruitment and scheduling process, while Figure 6 describes the explicit hair preparation options during signal optimization.

#### 2.4.1 Before Visit: Staffing

To optimally establish trust with Black participants, it is essential to employ research staff with lived experience, which in this context means having Afro‐textured hair, as well as knowledge and expertise of styling Afro‐textured hair. This person or persons may either be a member of the research team already or be someone hired specifically for this purpose, and they may be a professional hair stylist or an amateur/someone with ad hoc experience. We refer to this individual(s) as the “Hair Equity Specialist.” For the present study, the Hair Equity Specialist was author A.S.—an undergraduate research assistant hired specifically for this role. In addition to the Hair Equity Specialist, each visit was staffed by at least one other researcher with expertise in fNIRS setup protocols.

#### 2.4.2 Before Visit: Hair Equity Screener

Based on the reviewed literature, author A.S.’s lived experience, and our team's expertise in working with children and families, we developed an intake form that we refer to as our “hair equity screener” to send to participants (or pediatric participants’ caregivers) who would be engaging in fNIRS studies that require whole‐head capping (i.e., this may not be necessary if studies only use prefrontal montages that only touch the forehead). Note that the present version of this screener is designed for a study of preschool children aged 3–5 years (the full text of the screener is available in Supporting Information S3). It begins with a personal introduction to the Hair Equity Specialist to help establish trust. Alongside a photo of herself, she describes her Black identity and her experience styling her own natural hair since she was 13 years old. She continues, “I hope that seeing someone who looks like you will make you more comfortable with your child being in our study. Below are more specific questions on how you take care of your child's hair.” Anecdotal participant feedback expressed gratitude for the humanizing personal details about the research team and indicated that these details have been incredibly helpful in establishing trust, providing the foundation for a relationship between participants and researchers, and offering families background information that can help ease pediatric participant anxiety through preparation before the visits.

The screener then asks several open‐ended questions about the child's hair care routine, including where the caregiver shops for hair care products, the frequency of wash days, and what a normal wash day procedure involves. Next, we ask whether the child requires distraction, such as watching television, during the hair styling process. On our end, this information allows us to prepare a preferred program or activity that we can have readily available so that the child can be comfortable and engage in as familiar a process as possible.

Afterward, we asked caregivers to report whether or not their child is tender‐headed. Tender‐headed children are especially sensitive to their head, hair, and scalp being touched, and this sensitivity demands extra care and attention in the hair care and styling process. For some children, or even adults, tender‐headedness is associated with a nervousness or unease about styling their hair, so collecting this information is essential for the visit planning process to ensure both participant comfort and hair preparation for signal acquisition.

Next, we asked about specific products that the caregiver uses in the child's hair, including shampoo, conditioner, and any styling products (e.g., gel, cream), as well as whether or not they ever use heat (e.g., blow‐dryer). We collected information about curl type and texture and hair porosity by linking participants to an online quiz and descriptions to help them determine their types (Carol's Daughter 2023; Viola n.d.). Finally, we provided an open‐field response for anything else the caregiver may want to share about their child's hair, routine, personality, or other factors. In total, this intake form establishes trust between the participant and the research team while also allowing the researchers to adequately prepare for the participant.

#### 2.4.3 Before Visit: Scheduling the Visit

If possible, participants were scheduled to come on or just before a wash day, when their hair was planned to be natural and loose. However, five adult participants were purposely invited to come while they had a protective style in place, in order to investigate possibilities for working around the style (see Table 1). After a participant completed the intake form, we scheduled the participant's lab visit. Notably, these visits typically took longer than visits for participants who do not have Afro‐textured hair. While the exact timing depends on the specific montage being used, the participant population, and their tolerance for sitting still (e.g., children, individuals with disabilities), we planned for an extra hour and compensated participants for the full time they spent in the lab. In Supporting Information S4, we provide examples of infographics we provide to participants on what to expect during their lab visit.

#### 2.4.4 Before Visit: Materials Used

We acquired a group of hair tools and products commonly used on Afro‐textured hair to assist with hair preparation, including hair pics, mini elastic hair ties, scrunchies, rat tail combs, wide tooth combs, metal or plastic alligator clips, hairdryer with attachment, hair gel, hair cream, leave‐in conditioner, spray bottle, barbicide, and disinfectant jar. In addition, we also used some materials specifically for fNIRS preparation, including washable chalk markers, light‐up crochet hooks, clamps, and foam mannequin heads. The use of these materials is detailed below. Further, in Supporting Information S1, we provide a “shopping list” of all materials we currently keep on hand, details of how and why we use the products, and suggestions for where to purchase these items.

#### 2.4.5 During Visit: Pre‐Preparation Procedures

After providing informed consent (and child assent), participants were provided with a “distraction” of their choice—typically, children watched YouTube videos on an iPad, while adults used their cell phones. We first took a series of photographs of the participant's existing hair from the front, back, sides, and aerially from above, to capture dimensions of the hair and how it may differ across the scalp. We also recorded the participant's skin color, hair color, hair type, and hair style.

A researcher then measured the circumference of the participant's head and populated the corresponding EasyCap with the optode montage in default tensions (described above). It was not uncommon to need to use a larger size cap (compared to same aged participants without Afro‐textured hair) to accommodate the hair volume. Next, we placed the cap on the participant for initial signal optimization. We found it helpful to have two people participate in the capping process: one researcher held the front of the cap in place on the participant's forehead, while the other researcher pulled the cap over the participant's head and hair from behind. We then ensured that the cap was properly positioned and the sensors were making as much scalp contact as possible without engaging in an extensive intervention (e.g., for many White participants, all that is needed for an excellent signal is to shimmy the cap back and forth to let the optodes comb through the hair). Next, we ran a baseline signal optimization, using Aurora version 2021.9.0.6 on a Dell laptop running Windows 10, which took about 30–60 s. We documented the baseline signal optimization by taking a screenshot.

Both researchers then discussed appropriate intervention steps to improve signal quality. With adult participants, we discussed the proposed next steps with them, confirmed their comfort with the processes, and answered any questions. With pediatric participants, we first discussed the processes with the adult guardian. We then used developmentally appropriate language to explain that our Hair Equity Specialist will do their hair, and we obtained children's verbal assent to continue.

#### 2.4.6 During Visit: Hair Preparation Procedures

Below, we describe a “menu” of techniques that can be employed to increase the fNIRS signal with Afro‐textured hair, ranging from least to most involved. These procedures were developed for use with NIRx NIRSport 2 hardware and software; however, they should be generally applicable to any fNIRS setup. In general, we aimed to use the least invasive process that achieves a good quality signal (see Figure 6 for a flowchart). Different participants required different approaches based on their hair type, their willingness and tolerance for hair styling, and the specifics of the fNIRS montage being used. Some participants required a trial of multiple approaches iteratively to achieve the best result. However, throughout the hair preparation procedure, the participant's comfort and hair integrity were prioritized, and the researcher engaged in an active assent process with each additional step or strategy attempted.

Interventions were applied to the whole head for child participants, as they were participating in an existing study. However, for all adult participants, we only applied the intervention to the right side of the head and kept the left side as is, so that each participant could serve as their own control. Further, the intentional inclusion of five adult participants with existing protective styles allowed us to test our full range of potential intervention options. The exact interventions used are detailed in Table 1.

##### 2.4.6.1 Option #1: Gently Move the Hair

Regardless of the hairstyle that the participant arrived wearing (natural, protective style, relaxed, or other), our first approach was always to test whether we could simply gently move the hair to allow the optodes to lie flat on the participant's scalp. The cap with pre‐populated optodes was fitted over the participant's head, and signal quality was monitored in real time to determine how interventions are affecting signal quality. Optodes or channels that did not have an excellent signal were flagged, and a researcher gently slid an LED‐tip crochet hook (see Supporting Information S1) into a nearby, unused slit in the cap and moved the hair out of the direct path of the optode to allow the optode tip to reach the scalp. Occasionally, researchers would also try pulling the hair through nearby slits/holes in the cap (as would be done with a highlighting cap) to help keep hair out of the way. This intervention involves minimal discomfort, as the overall process is minimally invasive and the tips of the LED crochet hooks are blunted plastic.

##### 2.4.6.2 Option #2: Use Pressure to Push Optodes Closer to the Scalp

For most individuals with Afro‐textured hair, the texture and curl pattern of the hair result in more volume of hair than thinner or straighter hair. Thus, even if hair can be successfully pulled away from the optodes, the volume of hair may still push the cap and optodes away from the scalp. To counteract this, we increased the tension of the spring‐loaded grommets, especially in the more posterior regions of the head, where hair tends to be thicker. We also often used a chin strap and/or chest strap to pull the cap downward and/or placed a beanie cap over the tops of the optodes to push them downward (in both cases, the researcher must ensure that the optodes are still perpendicular to the scalp). While these strategies were often very effective, they also occasionally increased participant discomfort if the optodes were pushing on the scalp too hard or the straps/over‐caps were too tight. For this reason, researchers checked on participant comfort both as adjustments were being made and also throughout the experiment (what was comfortable at the beginning may be uncomfortable by the end).

##### 2.4.6.3 Option #3: Gel the Hair

If the participant does not come to the lab with an existing protective style and they are wearing their hair naturally, our next course of action involves applying gel to see if that will hold the hair in place. We have found that this technique has been most effective for participants with shorter hair (i.e., less than 1 inch). The researcher will first wet the participant's hair significantly, then warm up the gel between their two palms, and then apply gel to regions where optodes will be, while pushing excess hair toward a region where optodes are absent (if possible).

##### 2.4.6.4 Option #4: Gather and Pin Large Sections of Hair

If the participant had very long, natural hair or had an existing protective style (e.g., box braids, mini twists) that could not be removed, our next approach was to gather and pin the hair away from the optodes in large sections. This technique involved using elastic hair ties and/or alligator clips to tie or pin excess hair toward a region without optodes (if possible). Additionally, existing braids, locs, or twists could be combined into larger braids. However, if there was too much volume pushed toward a single location, it could prevent the cap from lying flat against the scalp and reduce tension, so the hair was diverted to multiple other locations.

##### 2.4.6.5 Option #5a: Strategically Part and Pin/Tie Small Sections of Hair

If a participant had natural, loose hair that was at least an inch long, a moderately intensive intervention was to strategically part the hair around the optode locations and pin or tie the hair in small sections. We started by using a rat‐tail comb to part the participant's hair straight down the midline from forehead to the nape of the neck (the “longitudinal” line) and fastened each side with alligator clips. Then, we placed a measuring tape along this line from nasion (the distinctly depressed area between the eyes, just above the bridge of the nose) to the inion (base of the prominent occipital bump at the back of the skull) and marked the centermost point (location Cz in the international 10–20 system) using a washable chalk marker (white or other light/bright colors were most visible on dark skin/hair). The Hair Equity Specialist also showed the participant how easily the marker washed off by first dotting it on their skin, in order to mitigate anxiety about having the marker in one's hair.

From there, we marked points on the head where optodes were intended to be, using the tape measure and head size as a guide to the standard locations in the 10–20 system. For example, locations Fz and Pz should each be 20% of the longitudinal nasion–inion length away from the Cz point, and Fpz and Oz an additional 20% from those. Specific to our montage, we made two marks anterior to Cz and two marks posterior to Cz, each 10% of the nasion–inion length from the prior dot (note that the montage did not have very posterior optodes, and the anterior‐most optodes tended to lie on the forehead with no hair to move). We then used the rat‐tail comb to firmly part vertical lines in the hair where optodes would lie on the scalp and used elastic hair ties or small clips to tie off the sections.

##### 2.4.6.6 Option #5b: Strategically Part and Braid/Twist Small Sections of Hair

**FIGURE 7:**
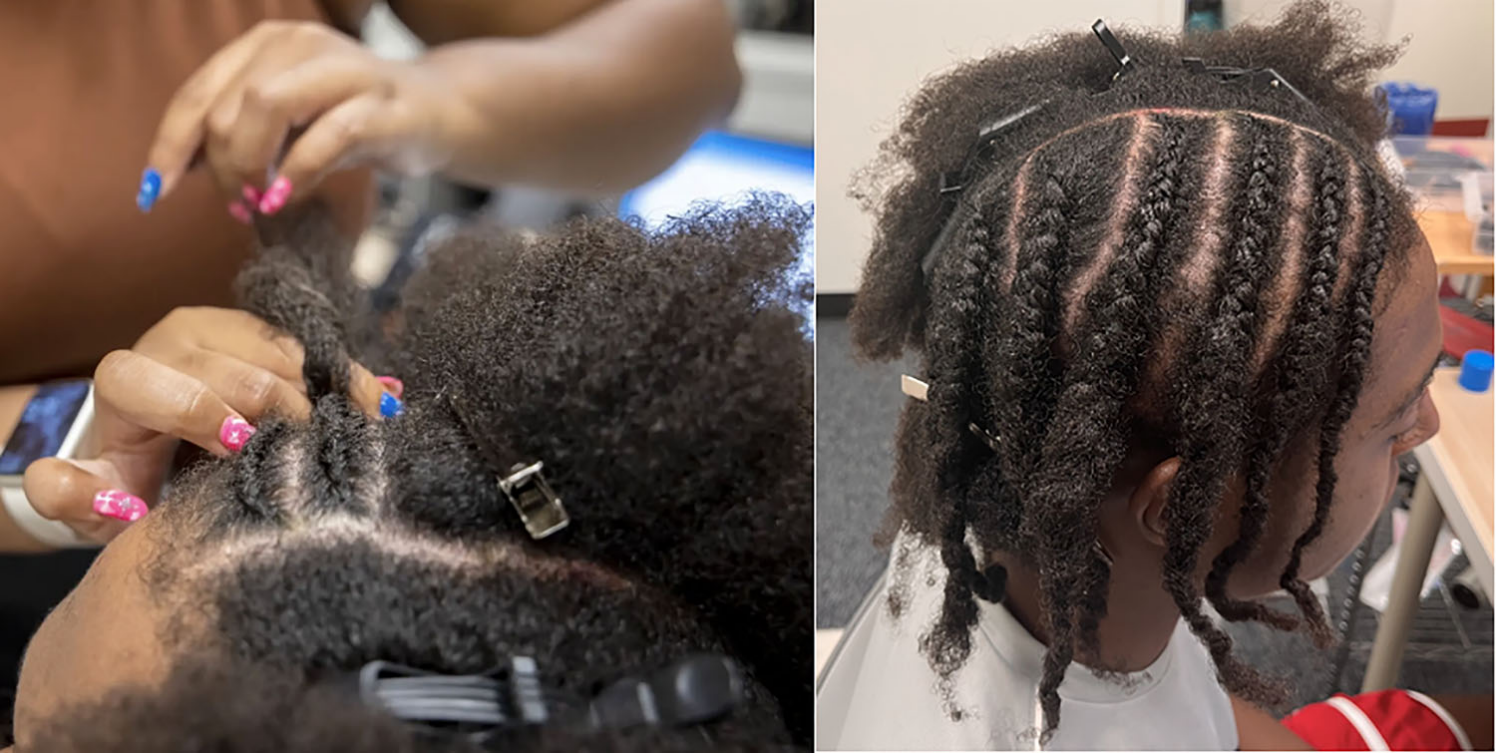
Left: Cornrowing the right side of the participant's head. Right: Completed cornrows to match study‐specific optode placement.

When a participant had longer hair, tying or pinning the hair sections often did not sufficiently keep the volume of hair away from the parts. In this case, we parted the hair in the same way as Option 5a and then either cornrowed or flat‐twisted the hair sections as flat to the scalp as possible (Figure 7). Specifically, we started cornrows or twists near the optode‐dense regions and worked away toward regions without optodes, since the cornrows/twists became fatter as more hair was added and thus could push the optodes away from the scalp. While this was the most time‐intensive hair preparation option (it often took 30 min to an hour), it was also the most durable design, which may be preferred for longer studies where the fNIRS needs to be worn for a longer duration.

##### 2.4.6.7 Option #6: Have the Participant Strategically Part and Braid/Twist the Hair at Home

**FIGURE 8:**
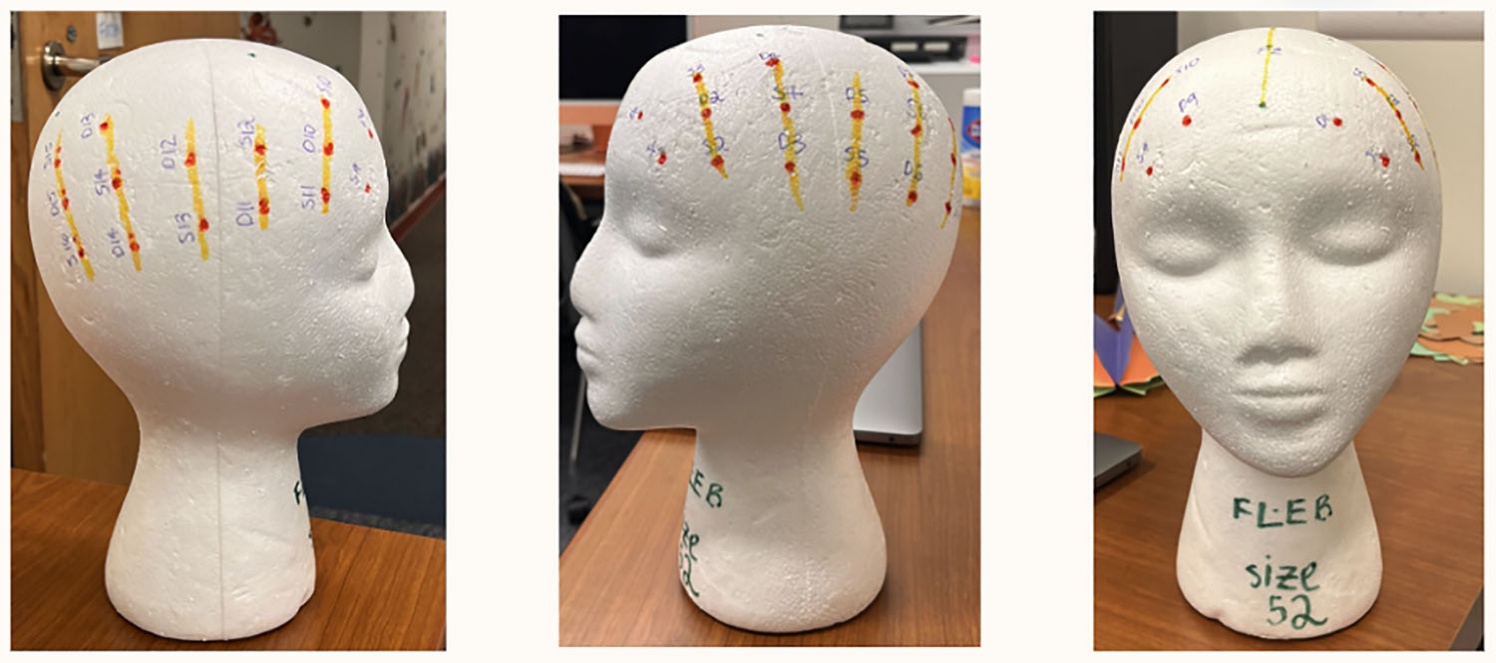
Mannequin head annotated with the measured midline landmarks (green dots in rightmost picture), study optode locations (red dots), and cornrow locations (yellow lines). The annotated mannequin head size is 52 cm.

For one child participant who was extremely tender‐headed and apprehensive about having a stranger work with their hair, we instructed the participant's parent to create the study‐specific braiding at home or at a salon before coming for their research visit. While this limited the precision that could be achieved by the experienced researchers, it was deemed the only option to include this participant. The Hair Equity Specialist set up a video call with the child's parent to describe options, demonstrated where the parts would be on a mannequin head (Figure 8), described the measurements, and showed examples of prior participants (faces obscured). Ultimately, this resulted in partially usable data, as some braids were aligned with the montage, but some were slightly misaligned, such that the optode landed on top of a braid rather than a part. Researchers did not attempt to rebraid the hair.

#### 2.4.7 During Visit: Realtime Signal Evaluation

Once the hair intervention was completed, we ran another signal optimization and took a screenshot. If additional interventions were needed, we repeated the process and took a final optimization screenshot after all interventions were complete. The total length of the hair intervention ranged from 30 min to 3 h. We also took photographs of the participant wearing the cap and additionally took a 3D whole head image using a Structure Sensor Pro (Occipital 2022) to use for post hoc evaluation of optode locations. Lastly, we removed the cap and took a final set of photographs (front/back, sides, and aerial) to document the final hair equity intervention procedure for recordkeeping. In addition to these photographs, we also kept written records of the participants’ skin color, hair color, hair texture, and hair style.

#### 2.4.8 After Visit: Exit Interview

After each study session, we conducted exit interviews with participants to learn more about their experiences with us throughout the session, with the aim of incorporating the feedback and continuing to improve our approach. We inquired about how comfortable participants felt about their hair being touched, the hair care products we used, and the intervention(s) conducted with their hair. Interviews were audio‐recorded for future reference. Finally, participants were compensated for the total amount of time they spent in the lab, including the hair equity prep.

### 2.5 Analyses

Improvement was quantified in multiple ways to account for the variation in the available data across participants.

First, while all participants had data from the postintervention time point, only 13 of 18 (72%) participants also had data from the preintervention time point. As noted above, the process of creating these practices was iterative, and we did not have the foresight to collect preintervention data from the first three adults and two children. Thus, reported pre‐ to postintervention analyses only include *n* = 13 participants (eight adults, five children). These analyses met assumptions for parametric tests, so paired *t*‐tests are reported.

Additionally, all adult participants (*n* = 11) had interventions done only to the right side of their heads, so that they could serve as their own controls. However, because child participants were enrolled in a larger longitudinal study, interventions were completed on their whole head to maximize data quality. Thus, for pre‐ to postintervention analyses, we always analyze the total number of channels that received intervention (whole head for children, half of the head for adults). Additionally, we also report the comparison between the postintervention left and right sides of the head for the 11 adults who served as their own control. These analyses did not meet normality assumptions, so nonparametric Wilcoxon signed‐rank tests are reported.

## 3 Results

**FIGURE 9:**
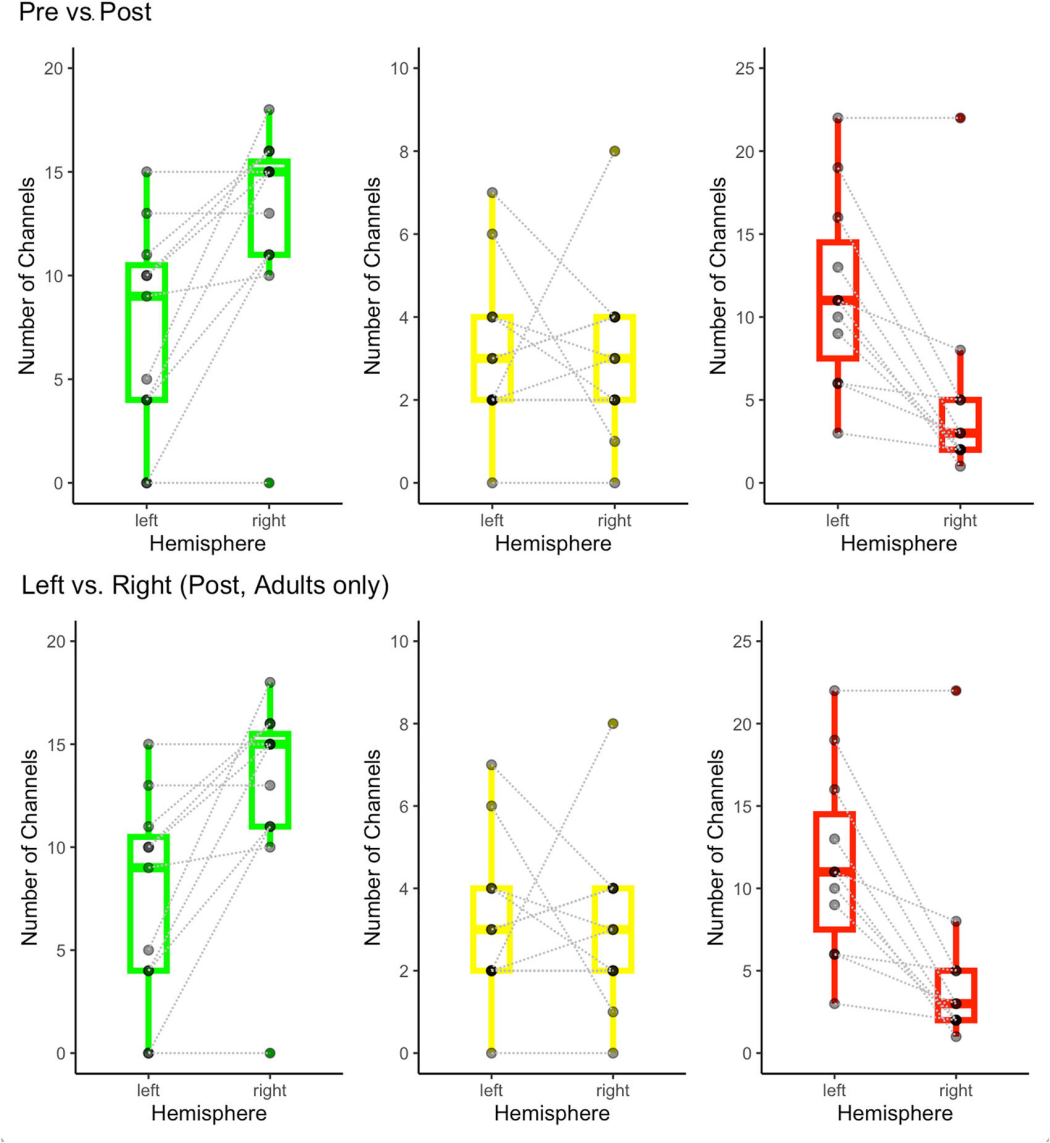
Change in signal quality classification (i.e., green, yellow, and red) of regular distance channels. Top: Change in signal from preintervention to postintervention, across all channels intervened upon. Bottom: Difference in signal between right (intervened) and left (non‐intervened) side of the head at postintervention, for adult participants.

From preintervention to postintervention (Figure 9, top), there was a significant increase in the number of green channels (*t*(12) = 4.58, *p* < 0.001; mean increase = 5.85 channels) and a significant decrease in the number of red channels (*t*(12) = −5.79, *p* = 0.001; mean decrease = 5.69 channels). There was no significant change in the number of yellow channels. Among participants who had intervention applied to only half of the head (Figure 9, bottom), the intervened side (vs. non‐intervened side) had significantly more green channels (*W* = 0.0, *p* = 0.014; mean difference = 5.36 channels) and significantly fewer red channels (*W* = 55, *p* = 0.006; mean difference = 6.45 channels). There was no significant difference in the yellow channels between the intervened and non‐intervened sides. All four significant results survived FDR correction for multiple comparisons (all adjusted *p* < 0.03).

To facilitate a qualitative comparison across individual participants, we calculated the percent improvement in usable channels (green or yellow; see Table 1). From pre‐ to postintervention, improvement in usable channels ranged from 0% to 150%, with an average of 50%. Comparing right to left, improvement in usable channels ranged from −5% to 59% (note that the participant with fewer usable channels on the right/intervened side used the montage with two fewer data channels on the right). Although the sample is not large enough for formal subgroup analysis, it appears that signal improvement was greatest for participants who came with loose hair that was cornrowed, and improvement was minimal for participants with protective styles (e.g., box braids and locs). Additionally, while it appears that improvement was greater for adults versus children, children tended to have better baseline signal quality before intervention, leaving less room for improvement. Finally, visual inspection suggests that the greatest signal improvement was seen in more anterior regions, as compared to more posterior regions, where hair is often thicker.

## 4 Discussion

The present study aimed to establish more inclusive practices for fNIRS research with participants who have Afro‐textured hair. We developed a series of culturally responsive procedures to improve signal quality while prioritizing participant comfort and hair integrity. These included hiring staff with lived experience of having and styling Afro‐textured hair, deploying an intake survey to mutually inform participants and researchers, strategically scheduling studies around participant wash days, and implementing a series of potential interventions, such as using gels and clips to shift hair, braiding or cornrowing around optode montages, and increasing tension on optode arrays. These practices led to an average 50% improvement in the number of usable channels, resulting in all but one participant meeting conventional inclusion criteria.

### 4.1 General Recommendations for fNIRS Researchers Working With Afro‐Textured Hair

Building on our experiences and findings, we offer several broader recommendations for researchers conducting fNIRS studies with participants who have Afro‐textured hair. First, whenever possible, research teams should hire and meaningfully involve staff with lived experience of having, caring for, and styling Afro‐textured hair. Such individuals may be students, community members, or professional stylists, and their involvement can be critical for establishing trust with participants, reducing anxiety, and minimizing unintentional harm to participants’ hair, comfort, and sense of dignity. Having individuals on research teams who hold similar identities to their participants can help ensure participants feel as comfortable as possible and may also allow participants to voice concerns with diminished fear of dismissal due to racial bias (Nicolaidis et al. 2010). Although non‐Black researchers can learn hair‐equity techniques, shared lived experience often facilitates communication and rapport in ways that technical training alone cannot. Importantly, whoever fills this role should be compensated appropriately for their time, expertise, and labor, with compensation structures that may reasonably differ depending on whether this individual is a student research assistant, a community consultant, or a professional stylist.

Researchers can identify and recruit such individuals by tapping into their surrounding communities, both within and outside of the research institution. For teams based at universities, this may include advertising positions through academic programs, majors, and minors, and sharing job postings with undergraduate program directors, academic advisors, and departmental staff. In addition to commonly targeted departments such as Psychology and Neuroscience, researchers may consider outreach to departments focused on societal inequities (e.g., African‐American Studies, Sociology), as well as student clubs and organizations such as Black Student Unions, First Generation Student Associations, and cultural dance teams. Finally, partnerships with Black hair professionals from the local community may represent another valuable avenue for identifying individuals with relevant expertise and lived experience.

Second, intake or screening surveys should be tailored to the specific participant population and study design. Our hair equity screener was developed for preschool‐aged children and their caregivers, but other studies may require modified questions that better capture relevant hair care routines, styling practices, durability needs, and participant preferences. Researchers are encouraged to view such screeners as living documents that evolve alongside the study and participant feedback, rather than as static forms.

In addition to informing preparation procedures, information gathered during intake can guide scheduling decisions that meaningfully impact data quality. In the present study, we observed the greatest improvements in signal quality among participants who arrived with loose, natural hair that could be fully manipulated to accommodate the study‐specific montage. Accordingly, whenever possible, scheduling sessions near a participant's wash day—when hair is already planned to be taken out of protective styles—may allow for maximum control over hair preparation and, in turn, greater signal improvement. Note, however, that this approach may not be feasible for participants with long‐term protective styles, yet we recommend that researchers refrain from asking participants to remove protective styles solely for research participation without prior discussion, planning, and consent, given the immense time and monetary investment involved in creating such styles. However, if scheduling a participant to come in with loose hair is possible, transparent communication about scheduling preferences can help align research needs with participant routines and autonomy.

Third, one of the most salient takeaways from our investigation is that there is no “one size fits all” solution for conducting fNIRS research with participants who have Afro‐textured hair. While some participants required very little intervention (e.g., minimal pinning and/or gel application), others required much more extensive approaches (e.g., whole‐head cornrows). Research teams should therefore be prepared to iteratively try multiple techniques, beginning with the least intensive and progressing to more involved procedures as needed, while engaging in active informed consent and assent throughout the process. These procedures will almost certainly require more time than those used for participants without Afro‐textured hair; accordingly, researchers should plan visit lengths appropriately and compensate participants for the full time they spend in the lab. This, in turn, has broader implications for funding agencies, ethics review boards, and other stakeholders, who should recognize and support the additional time, staffing, and resources required to conduct fNIRS research in a truly inclusive manner.

Relatedly, we strongly recommend that research teams develop their own study‐specific procedures and decision trees for hair preparation and interventions. While we have described preparation steps specific to our montage, these steps may look different for studies targeting different brain regions or involving different participant populations. For example, parts may be desired in different locations or oriented differently (e.g., horizontal vs. vertical) depending on the optode layout and regions of interest. Additionally, longer studies with more tolerant adult populations may require more durable interventions (e.g., cornrows or twists), whereas shorter studies may succeed with minimal pinning or gel application. Explicitly mapping out these decision points in advance can help teams operationalize flexibility while still following a consistent, participant‐centered framework.

Across all stages of the research, meticulous documentation is essential. Researchers should carefully record details of the hair interventions used, participant comfort and tolerance, and any challenges encountered during setup. In addition, participant characteristics that may systematically influence signal quality—such as skin tone, hair color, hair texture, hair density, and hairstyle—should be documented whenever possible and considered as potential covariates in analysis. Such reporting not only improves transparency and reproducibility but also allows the field to more accurately quantify and address sources of bias in fNIRS data.

Finally, we encourage the routine use of postsession exit interviews or debriefings. Soliciting participant feedback about comfort, communication, and hair care experiences provides invaluable information for refining procedures over time and signals to participants that their perspectives are valued. Continual refinement based on participant input is a critical component of truly inclusive research practice.

Though tool improvement has not been the focus of this investigation, we offer a few recommendations and ideas. First, the fNIRS cap design could better accommodate voluminous hair. For example, larger holes distributed throughout the cap could allow hair to be pulled through more effectively, enabling the cap to lie closer to the scalp. The optimal placement of such holes may vary depending on the study‐specific optode montage, suggesting that customizable cap designs would be ideal. Additionally, given the volume and density of Afro‐textured hair, it may be necessary to produce larger cap sizes overall. For instance, one commonly used brand, EasyCap (Brain Products), currently only extends to 64 cm in circumference, which may still be too restrictive for adults with very voluminous hair. Brush‐fiber–tipped optodes (e.g., Khan et al. 2012) may also help comb through dense or coily hair, and higher tension levels of spring‐loaded grommets could further improve scalp contact. However, we caution manufacturers to prioritize participant comfort to avoid introducing pain‐related inequities for Black participants.

Additionally, while we have focused primarily on signal attenuation due to hair, another potential source of bias in fNIRS data with Black participants is attenuation related to higher levels of melanin in the skin. Existing fNIRS technologies generally assume a fixed light pathlength through the brain across participants; however, because melanin is highly absorbent, individuals with darker, more melanated skin may experience systematic, nonlinear reductions in signal strength. This attenuation could lead to inaccurate estimations of relative changes in oxygenation in regions of interest (Kwasa et al. 2023; Wassenaar and van den Brand 2005). A full discussion of melanin‐related limitations and methodological solutions is beyond the scope of this paper, but readers are directed to prior and emerging work in this area (e.g., Bronkhorst et al. 2019; Patel et al. 2024; Roy et al. 2024; Sardar et al. 2001; Yücel et al. 2024).

### 4.2 Limitations and Future Research

This work is not without limitations. First, our sample size was small and did not evenly represent all subtypes of Afro‐textured hair, precluding rigorous subgroup analyses by hair type, texture, color, length, or style. As such, these findings should be interpreted as proof of concept rather than a definitive evaluation of technique efficacy across phenotypes. Future studies with larger and more diverse samples are needed to systematically examine which interventions are most effective for specific hair characteristics.

Second, adult participants were recruited from the social networks of author A.S., which may have increased their willingness to engage in more intensive hair interventions. While this highlights the central role of trust in research participation, it may limit generalizability to participants without pre‐existing relationships with the research team. Additionally, our approach of using adult participants as their own control by only intervening on one side of the head may have ultimately decreased optode–scalp contact on the intervened‐upon side, due to the natural lift of hair on the control side. However, this likely *under*estimates the effectiveness of the intervention, so signal improvement might be even better with whole‐head interventions.

Third, our primary outcome relied on categorical signal quality classifications (red, yellow, green) rather than continuous metrics (such as coefficient of variation), because these are the measures on which users will ultimately base inclusion/exclusion decisions. For example, one could observe statistically significant improvements in numerical signal quality, but if the channel is still coded as red (“unacceptable”), then the data are still likely not usable. Because of this decision, we did not retain the numerical calibration files (only screenshots of the visual calibration) and are unable to quantify improvement in terms of these more precise metrics. Additionally, we did not assess signal stability over the duration of a full experimental session for adult participants, nor did we collect posttask calibrations for children, limiting conclusions about the long‐term durability of the interventions. Future research should examine how interventions influence more fine‐grained measures of signal quality and the stability of these changes across a full study.

Future research should also evaluate the combination of advances in hardware design, signal processing, and transparent reporting standards with participant‐centered, culturally responsive hair preparation practices, in order to most comprehensively reduce compounded sources of bias.

## 5 Conclusion

In conclusion, this investigation aimed to develop and evaluate best practices for conducting fNIRS research with participants who have Afro‐textured hair. Without efforts at multiple system levels to diversify the research workforce, renounce scientific racism and exclusion, and repair damaged relationships with minoritized communities, calls to simply diversify samples fall short (Murray et al. 2021; Varma et al. 2021). As such, we hope that this investigation opens conversations about research practices at several levels of analysis.

## Funding

This work is supported by the National Institute of Child Health and Human Development (grant R00‐HD103873 to R.R.R.).

## Conflicts of Interest

The authors declare no conflicts of interest.

## Supporting information



Supplemental Materials

## Data Availability

The data that support the findings of this study are openly available in OSF at https://osf.io/hsu45/.

## Endnotes

1 Note that there are myriad nontechnological reasons for the exclusion of Black individuals in human subjects research broadly, and in neuroscience research specifically. For a further discussion, please see Scharff et al. (2010) and Smirnoff et al. (2018).
