# Supplementary material for: Developing Best Practices for Inclusion in fNIRS Research: Equity for Participants With Afro‐Textured Hair
Source: Dev Psychobiol. 2026 Feb 5;68(2):e70134. doi: 10.1002/dev.70134 (PMC12877426; doi:10.1002/dev.70134)

**Supplemental Materials**

Supplemental Material 1. Hair equity tools

Following is the list of hair care products our lab uses to prepare participants’ hair for hair intervention. Please note that this list is not final and items can be added if/as needed depending on study design and intensity of hair intervention.

- Foam mannequin heads
- Light-up crochet hooks
- Barbicide
- Disinfectant jar
- Spray bottle
- Chalk markers
- Afro pick
- Mini elastic hair ties
- Scrunchies
- Rat tail combs
- Wide tooth combs
- Metal or plastic alligator clips
- Hairdryer with attachment (Kiss)
- Hair gel (Edge Booster)
- Hair grease (Blue Magic)

Supplemental Material 2. Effects of Intervention on Short-distance Channels

Fifteen participants (8 adults, 7 children) in the current investigation used optode montages that included 8 short-distance channels distributed across the scalp (see main text figure 7). Pre-post and left-right comparisons were evaluated with nonparametric Wilcoxon signed-rank tests.

There were no significant differences in the number of green or red channels from pre- to post-intervention, nor between the left and right hemispheres at post-intervention. There was a small significant decrease in the number of yellow channels from pre-to-post (W = 21, *p* = .019; mean increase = 0.55 channels), but this did not survive correction for multiple comparisons (adjusted *p* = 0.118).

However, even pre-intervention, the short-distance channels had reasonably good signal quality, with only one participant having more than 1 red channel at baseline. Thus, short-distance channels appeared to be less affected by Afro-textured hair than long-distance channels. However, this should be examined more systematically with large numbers of short channels placed in various positions across the scalp.


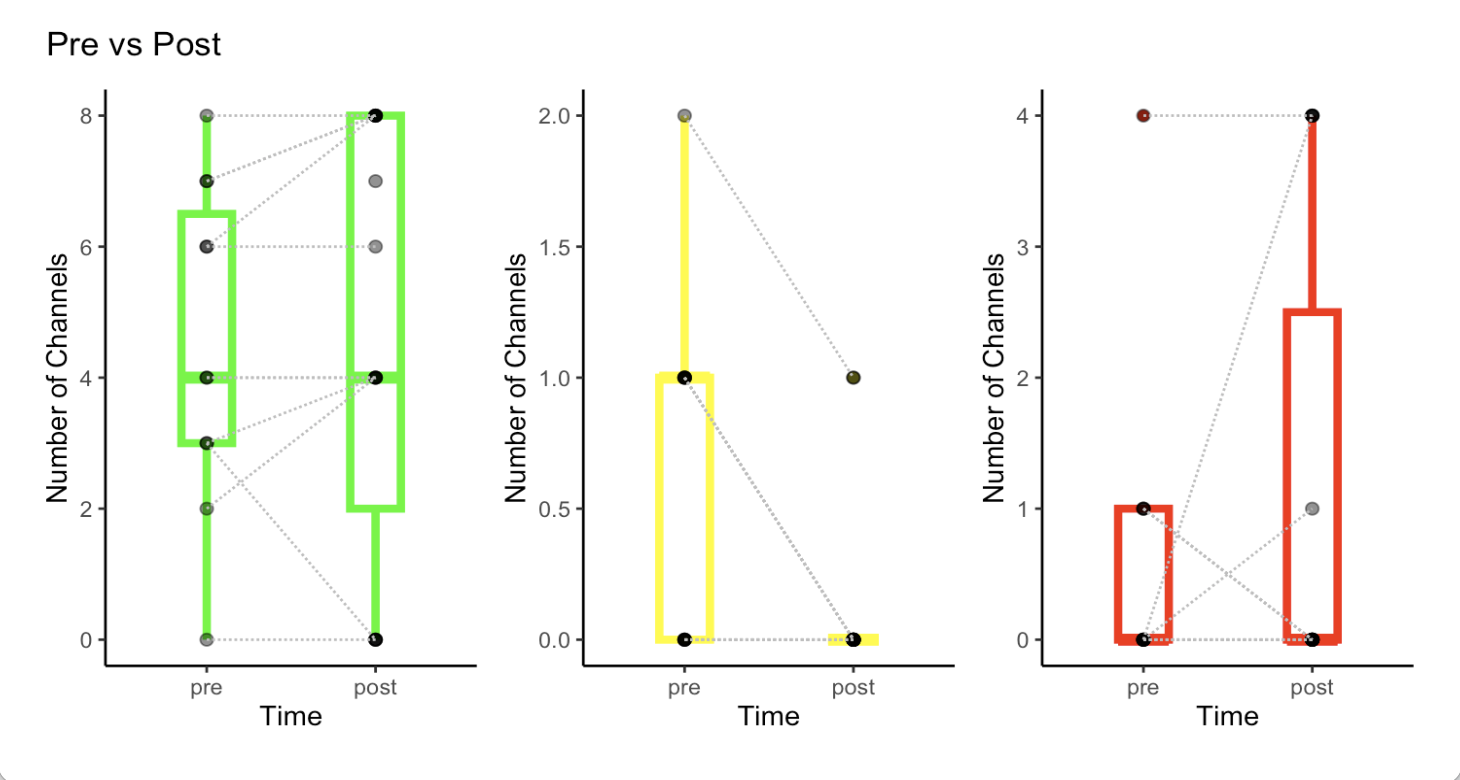


Supplemental Figure 1: Change in signal quality classification (i.e., green, yellow, and red) of short distance channels from pre-intervention to post-intervention, across all channels intervened upon (8 for children, 4 for adults).

Supplemental Material 3. Hair Equity Questionnaire

The following is a list of the questions we ask hair equity participants. These questions range from individuals' hair care practices, products used, and routines. Please note that these questions can be tailored to your specific study and can be as in depth/surface level as you would like them to be.

**Introductory text:**

This is the intake form for African American/Black families to fill out for their kids within our study. I myself am Black and have experience braiding and doing my own natural hair since I was 13 (for context I am 22). I hope by seeing someone that looks like you will make you more comfortable with your child being in our study. These are more specific questions on how you take care of your child's hair on a daily basis.

**Specific Questions for African American families and participants:**

1. Where do you regularly shop for your hair care products?
2. What does your wash day schedule look like?
3. Explain what a normal wash day looks like for you and your child.
4. Do you normally need to distract your child while you are doing their hair? (For example, when I was little and my mom would do my hair, she would sit me in front of the TV while she was doing my hair)
5. Please describe how you typically distract your child while doing their hair.
6. Is your child tenderheaded?
7. What products do you use to style your child’s hair? (maybe what shampoo and conditioner you use and one or two products you use when styling the hair)
8. Do you use heat in your child’s hair care routine?
9. Are you comfortable with me using a minimum amount of heat on your child’s head? I would just be blowing out the hair of the child just to stretch the curl pattern.
10. What type of porosity would you say your child’s hair has? What is their curl pattern type? (for instance I have a 4c curl pattern and low porosity curls. If you want you can take these two quick quizzes to find out this information.

Curl type Quiz- <https://www.carolsdaughter.com/curl-type-quiz.html>

Hair Porosity Quiz- <https://curlsmith.com/blogs/curl-academy/hair-porosity-guide#what>)

1. I want to keep the lines of communication open with you as the parent. Is there anything I should know about your specific child? Any tips or tricks that you use to make wash day go faster for you and your child.

Supplemental Material 4. Pediatric fNIRS & Hair Equity Infographic

The following is an infographic providing an overview of (1) pediatric fNIRS in one of our lab’s longitudinal studies of preschoolers and (2) the hair equity process for participants. The flyer is two-sided (front and back) and provided in both English and Spanish.


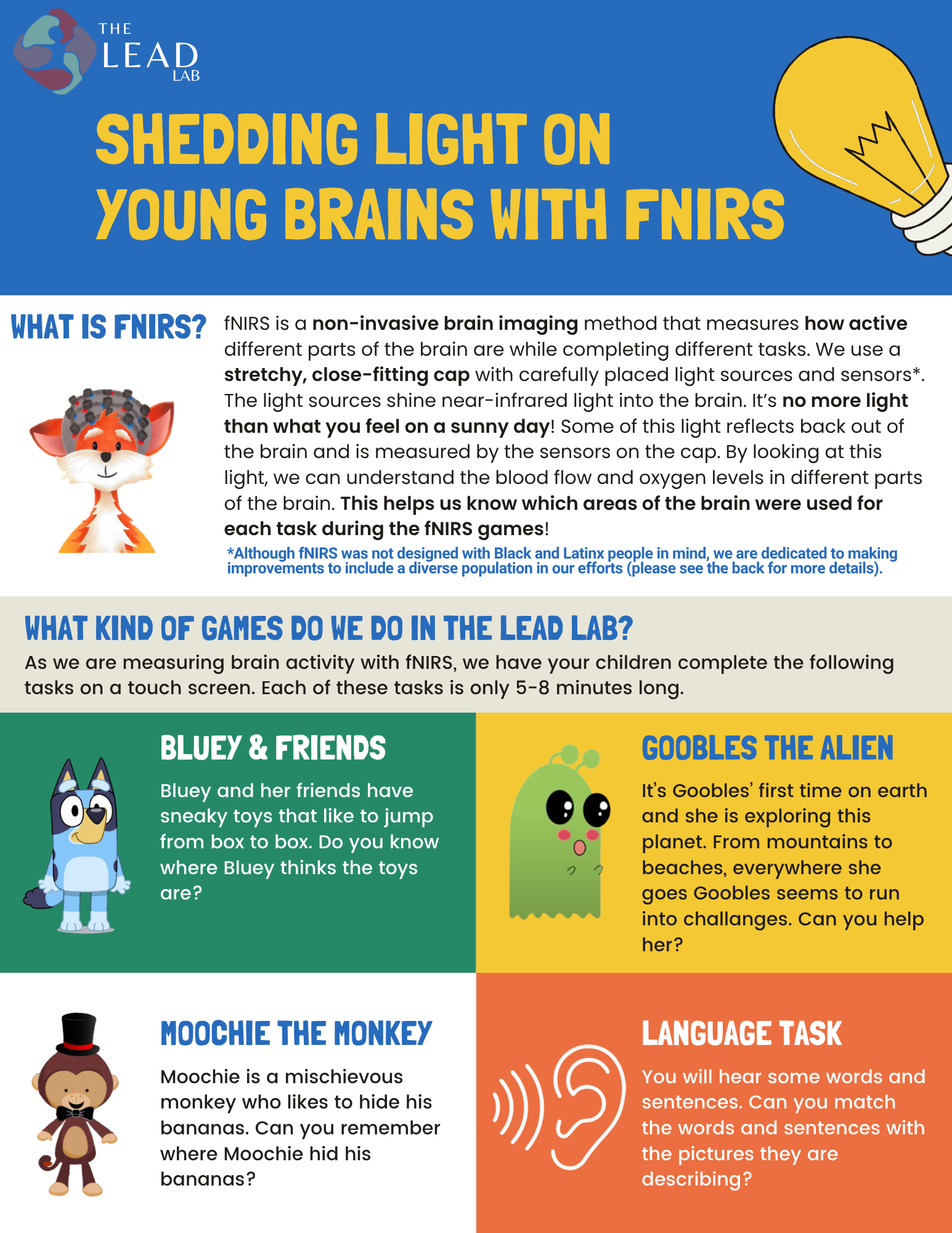


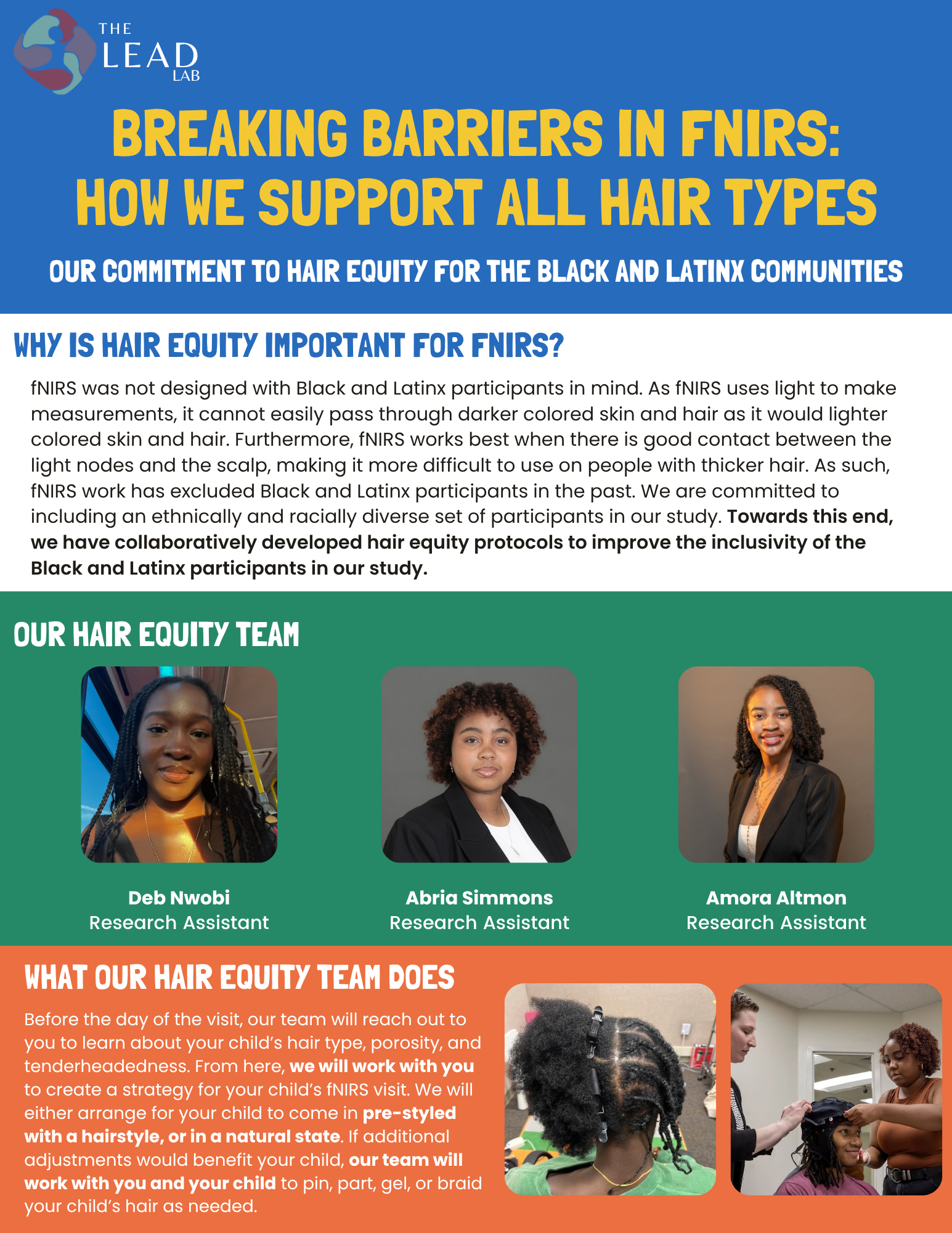


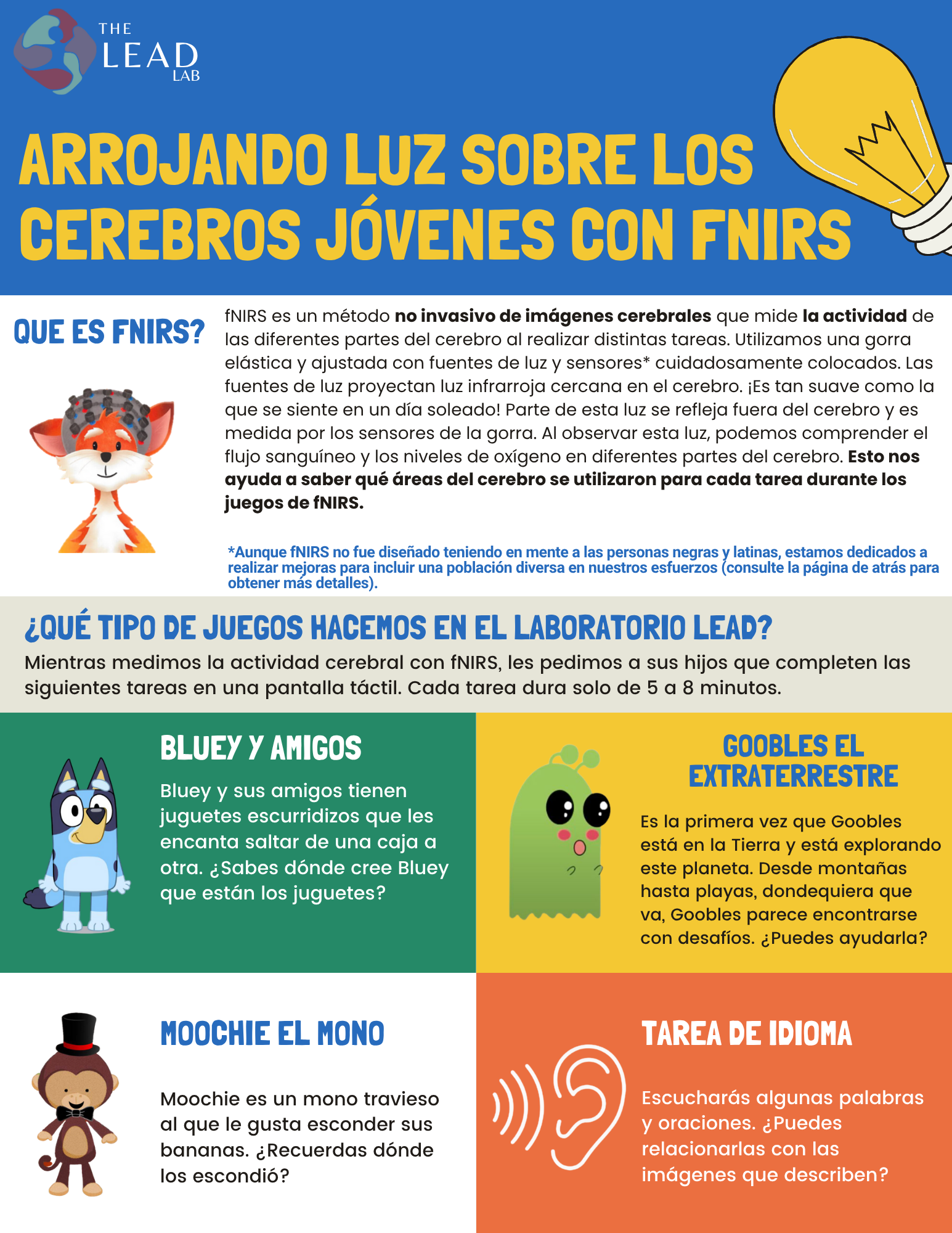


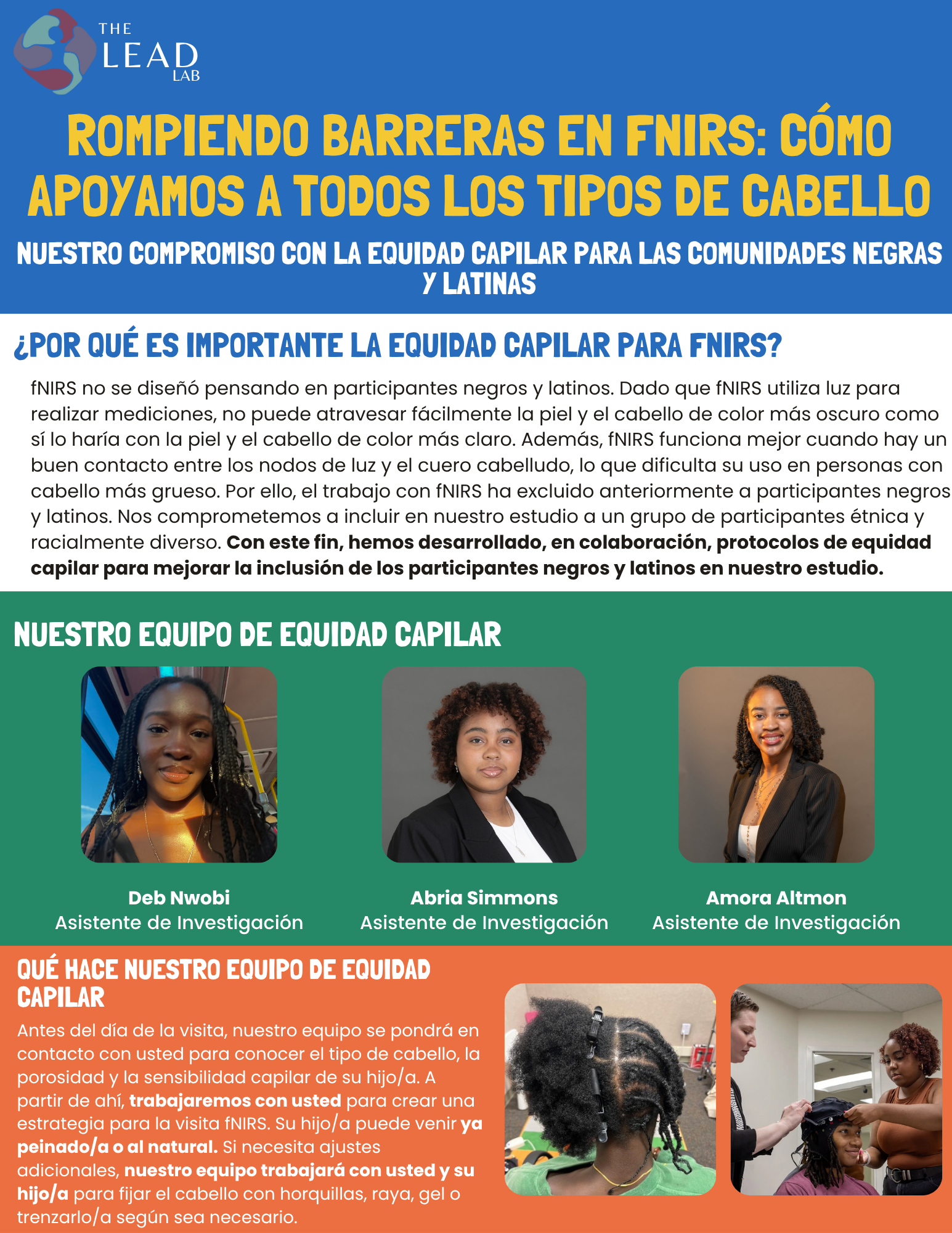

Supplement: Supplementary file 1 — Supplemental Materials [file DEV-68-e70134-s001.docx]
